# Sleep Promotes, and Sleep Loss Inhibits, Selective Changes in Firing Rate, Response Properties and Functional Connectivity of Primary Visual Cortex Neurons

**DOI:** 10.3389/fnsys.2018.00040

**Published:** 2018-09-07

**Authors:** Brittany C. Clawson, Jaclyn Durkin, Aneesha K. Suresh, Emily J. Pickup, Christopher G. Broussard, Sara J. Aton

**Affiliations:** ^1^Department of Molecular, Cellular and Developmental Biology, University of Michigan, Ann Arbor, MI, United States; ^2^Neuroscience Graduate Program, University of Michigan, Ann Arbor, MI, United States; ^3^Committee on Computational Neuroscience, University of Chicago, Chicago, IL, United States; ^4^Information Technology Advocacy and Research Support, College of Literature, Science and the Arts, University of Michigan, Ann Arbor, MI, United States

**Keywords:** *in vivo* recording, sensory cortex, excitability, information processing, signal-to-noise

## Abstract

Recent studies suggest that sleep differentially alters the activity of cortical neurons based on firing rates during preceding wake—increasing the firing rates of sparsely firing neurons and decreasing those of faster firing neurons. Because sparsely firing cortical neurons may play a specialized role in sensory processing, sleep could facilitate sensory function via selective actions on sparsely firing neurons. To test this hypothesis, we analyzed longitudinal electrophysiological recordings of primary visual cortex (V1) neurons across a novel visual experience which induces V1 plasticity (or a control experience which does not), and a period of subsequent *ad lib* sleep or partial sleep deprivation. We find that across a day of *ad lib* sleep, spontaneous and visually-evoked firing rates are selectively augmented in sparsely firing V1 neurons. These sparsely firing neurons are more highly visually responsive, and show greater orientation selectivity than their high firing rate neighbors. They also tend to be “soloists” instead of “choristers”—showing relatively weak coupling of firing to V1 population activity. These population-specific changes in firing rate are blocked by sleep disruption either early or late in the day, and appear to be brought about by increases in neuronal firing rates across bouts of rapid eye movement (REM) sleep. Following a patterned visual experience that induces orientation-selective response potentiation (OSRP) in V1, sparsely firing and weakly population-coupled neurons show the highest level of sleep-dependent response plasticity. Across a day of *ad lib* sleep, population coupling strength increases selectively for sparsely firing neurons—this effect is also disrupted by sleep deprivation. Together, these data suggest that sleep may optimize sensory function by augmenting the functional connectivity and firing rate of highly responsive and stimulus-selective cortical neurons, while simultaneously reducing noise in the network by decreasing the activity of less selective, faster-firing neurons.

## Introduction

Sleep is hypothesized to play a critical role in learning and memory, by facilitating long-lasting plastic changes in the strength of synapses and across networks (Aton et al., 2009a,b; Diekelmann and Born, 2010; Chauvette et al., 2012; Yang et al., 2014; Puentes-Mestril and Aton, 2017). Among the mechanisms by which sleep may promote information storage in the brain, general synaptic downscaling has been proposed as a possible mediator. In theory, widespread synaptic downscaling is proposed as a homeostatic response by which network excitability could be constrained and signal-to-noise ratios for neuronal firing could be improved following widespread synaptic potentiation associated with waking experience (Tononi and Cirelli, 2003, 2014). This idea is supported by biochemical and transcriptomic studies in rodents, demonstrating that cellular markers of neuronal activity and synaptic strengthening are increased in the forebrain after a period of wake, and decreased after a period of sleep (Cirelli et al., 2004; Mackiewicz et al., 2007; Vyazovskiy et al., 2008). However, recent studies suggest that these effects may vary between brain areas (Thompson et al., 2010; Delorme et al., 2018) and as a function of experience (Ribeiro et al., 1999, 2002; Ulloor and Datta, 2005; Aton et al., 2009a; Seibt et al., 2012). Thus it is unclear whether downscaling is a phenomenon associated with experience-dependent plasticity in neuronal circuits, such as are initiated by learning. In addition, it is unclear whether downscaling occurs during rapid eye movement (REM) or non-REM (NREM) sleep. For example, studies of cortical neurons have attributed decreases in firing to slow wave activity in NREM (Vyazovskiy et al., 2009), while studies of hippocampal neurons have shown firing increases across bouts of NREM, and rapid decreases across REM sleep (Grosmark et al., 2012). Moreover, it is unclear whether, or how, sleep-dependent downscaling would affect the response properties and information-processing capabilities of individual neurons. Recent data suggest that sleep-associated decreases in synaptic strength and neuronal excitability are heterogeneous, even within a given brain region. For example, only a subset of synaptic structures appear to be reduced in size in the cortex across sleep (de Vivo et al., 2017), and only a subset of cortical neurons show significant decreases in firing rate after sleep (Vyazovskiy et al., 2009). The idea that these changes are not uniform, but may preferentially affect a specific subpopulation of network neurons, is supported by recent studies of firing rate changes in rodent frontal cortex and hippocampus across bouts of sleep and wake behavior. For example, Watson et al. (2016) found that while most rat cortical neurons show firing decreases across bouts of REM sleep, only those neurons that have the fastest baseline firing rates show firing decreases in NREM sleep. Overall changes in firing across sleep periods (containing REM, NREM and microarousals) are opposite between higher firing neurons (which show net firing decreases) and sparsely firing neurons (which show net firing increases). Thus instead of uniformly decreasing firing rates, sleep seems to narrow the distribution of firing rates among cortical neurons (Watson et al., 2016). In contrast to what is seen in frontal cortex, firing rates among both interneurons and principal neurons in hippocampal area CA1 generally increase across bouts of NREM, and dramatically decrease across bouts of REM (Grosmark et al., 2012). Available data suggests that as is true for cortical neurons, these changes in firing across sleep differentially affect higher-firing and lower-firing neurons (Miyawaki and Diba, 2016).

Recent studies have also characterized neurons in sensory cortical areas based on how coupled their firing is to that of the population (Bachatene et al., 2015; Okun et al., 2015). So-called “choristers” fire in a manner which is tightly linked to spontaneous population-level activity, while “soloists” tend to fire independently from the population. In sensory areas, fast-spiking interneurons, and bursting pyramidal neurons, tend to fire as choristers, while non-bursting pyramidal neurons fire as soloists (Okun et al., 2015). Bachatene et al. (2015) also demonstrated that population-coupling strength of neurons in sensory cortex varies as a function of firing rate. Thus, the neurons on the lower end of the firing rate distribution appear to be comprised of soloists, while high-firing neurons are likely choristers (Bachatene et al., 2015). Critically, the relationship between neurons’ population coupling strength, their sensory response characteristics, and their information-carrying capacity remains a matter of speculation (Bachatene et al., 2015; Okun et al., 2015). While soloists may be able to respond very selectively and precisely to sensory stimuli, choristers’ firing appears to carry additional information regarding an animal’s behavioral state and other non-sensory factors (Okun et al., 2015). Thus two important unanswered questions are how sleep and wake states affect soloist and chorister populations, and how this might be relate to sleep-dependent plasticity in neural circuits.

Recent work from our lab has shown that mean firing rates are differentially affected by sleep in mouse primary visual cortex (V1), depending on prior visual experience. For example, we have shown that when mice are presented with a single-oriented grating over a prolonged period (several minutes to an hour), neurons in the lateral geniculate nucleus (LGN) of the thalamus, but not V1, show an enhanced firing rate response to grating stimuli of the same orientation (Durkin et al., 2017). Only after a period of subsequent sleep do V1 neurons undergo a similar orientation preference change, marked by increased firing responses to similarly-oriented gratings (orientation-specific response potentiation: OSRP; Aton et al., 2014; Durkin and Aton, 2016; Durkin et al., 2017). After a visual experience that induces OSRP, firing rates for V1 neurons increase across bouts of sleep, particularly across REM sleep (Durkin and Aton, 2016). Thus state-dependent changes in V1 neurons’ firing rates are functionally linked to sensory plasticity and may vary as a function of prior sensory experience.

Here, we aim to address how brain state-dependent changes in different neuronal populations may affect the basic function and information-processing capabilities of sensory cortex (Aton, 2013). We first assess how both spontaneous and visually-evoked firing rates of sparse- or fast-firing V1 neurons are affected by visual experience, across a period of subsequent *ad lib* sleep, or across a similar period with partial sleep deprivation. We then assess how these parameters are affected in neurons which fire in a manner that is either weakly or strongly coupled to V1 population activity. We also determine which neurons’ orientation preferences are most altered in the context of OSRP.

## Materials and Methods

### *In vivo* Neurophysiology

All mouse procedures were approved by the University of Michigan Institutional Animal Care and Use Committee. For chronic recordings, male and female C57BL/6J mice (Jackson) aged 1–3 months (an age range where OSRP is induced robustly by visual experience; Frenkel et al., 2006; Aton et al., 2014; Durkin et al., 2017) were implanted with custom-built drivable headstages (EIB-36 Neuralynx) under isoflurane anesthesia, using previously described techniques (Aton et al., 2014). For each mouse, two 200 μm-diameter bundles of seven stereotrodes each (25 μm nichrome wire, California Fine Wire; Grover Beach, CA, USA) were placed in right hemisphere V1 (0.5–1 mm apart), reference and ground electrodes were placed in left hemisphere V1 and cerebellum, respectively, and three electromyography (EMG) electrodes were placed in nuchal muscle.

Following surgical procedures, mice were individually housed in standard caging with beneficial environmental enrichment (nesting material, toys and treats) throughout all subsequent experiments. With the exception of OSRP or blank screen experimental days, during which room lights were kept off, lights were maintained on a 12-h:12-h light:dark cycle (lights on at 8 AM, lights off at 8 PM). Food and water were provided *ad lib* throughout all procedures. After 1–2 weeks of post-operative recovery, mice were prepared for chronic stereotrode recording in their home cage, which was placed inside a sound-attenuated recording chamber (Med Associates). Mice were tethered using a lightweight cable for neural recording, and were habituated to daily handling, restraint, and head fixation over a period of 5 days. During this time, electrodes were gradually lowered into V1 until stable neuronal recordings were obtained. Recording stability was defined by the continuous presence of spike waveforms on individual electrodes for at least 24 h prior to the onset of baseline recording. Signals from each electrode were split and differentially filtered to obtain spike data (200 Hz–8 kHz) and local field potential (LFP)/EMG activity (0.5–200 Hz). Data were amplified at 20×, digitized, further digitally amplified at 20–100×, and recorded using Plexon Omniplex software and hardware (Plexon Inc., Dallas, TX, USA). For all chronic recordings, single-unit data was referenced locally to a recording channel without single-unit activity, to eliminate low-frequency noise.

### Visual Stimuli, OSRP Induction and Assessment of Visual Response Properties

A continuous 24-h baseline recording was carried out for each mouse, starting at lights-on (8 AM; CT0—Circadian Time 0). The following day at CT0, mice were head-fixed. To assess baseline (AM) visual response properties in V1 neurons, phase-reversing oriented gratings (spatial frequency 0.05 cycles/degree, 100% contrast, reversal frequency 1.0 Hz) of four orientations (0, 45, 90 and 135 degrees from horizontal) and a blank (dark) screen (to assess spontaneous activity) were presented to the left (contralateral) visual field. Each of these stimuli was presented eight times (10 s for each presentation) in a random, interleaved fashion. Neuronal firing rate responses were quantified and averaged for each stimulus orientation (and blank [dark] screen) across total presentation time (i.e., 10 s × 8 repetitions). Immediately following this baseline (8 AM; CT0) test, either a single grating stimulus (of a randomly-selected orientation) or a blank [dark] screen was continuously presented over a 30-min period to induce OSRP. Mice were then returned to their home cage and recordings continued until CT12 in complete darkness (with far-infrared illumination only, to prevent additional visual experience), at which time mice were again head-fixed for a second (PM) test of visual response properties. Between 30-min grating (or blank screen) presentation and PM testing, mice were either allowed to sleep *ad lib* (Vis Stim + Sleep: *n* = 14 mice, Blank Screen + Sleep: *n* = 7 mice), or were kept awake over the first 6 h (Vis Stim + early sleep deprivation [ESD]: *n* = 11 mice) or last 6 h (Vis Stim + late sleep deprivation [LSD]: *n* = 13 mice), using gentle handling procedures (Aton et al., 2014). Briefly, when mice were observed (under far-infrared illumination) taking stereotyped sleep postures and LFP data indicated transitioning from wake to NREM sleep, cages were tapped gently to awaken the mice. Later in the procedure (typically within the last 1–2 h), disturbance of the nest or lightly brushing the mouse with a cotton-tipped applicator was used to prevent sleep.

For each stably-recorded neuron (i.e., those with consistent spike waveforms on the two stereotrode channels across 24-h baseline recording, and across the 12-h experiment; see below for details of single-unit identification), a number of visual response properties were assessed during CT0 (i.e., the time of expected lights-on; “AM”) and CT12 (i.e., the time of expected lights-off; “PM”) tests (at 8 AM and 8 PM, respectively), using previously-described metrics (Aton et al., 2009a, 2013, 2014). Mean firing rates (in Hz) were averaged across all repetitions of the same visual stimulus (or blank [i.e., dark] screen). Mean blank screen firing (in Hz) was used as a metric of each neuron’s spontaneous activity. Mean firing at each neuron’s preferred stimulus orientation (i.e., that which evoked maximal firing rate response) was used as a metric of maximal visually-evoked firing rate. An orientation selectivity index (OSI45; used to indicate the strength of orientation tuning, regardless of orientation preference) was calculated for each neuron, as 1 − [(average firing rate at ±45° from preferred orientation)/(average firing rate at the preferred stimulus orientation)]. Thus OSI45 values for individual neurons range from 0 (minimal selectivity for the preferred stimulus orientation) to 1 (maximal selectivity for the preferred stimulus orientation). Neuronal visual responsiveness (to any visual stimulus) was assessed as a responsiveness index (RI), calculated as 1− [(average firing rate at blank screen presentation)/(average firing rate at the preferred stimulus orientation)]. RI values for individual V1 neurons typically range from 0 (not visually responsive) to 1 (maximally responsive), although negative values are possible for non-responsive neurons). Changes in these values between AM and PM tests (i.e., during the inactive phase of the rest-activity cycle; from CT0 to CT12) were assessed in non-sleep deprived and sleep deprived mice. For mice presented with a visual stimulus to induce OSRP, initial preference for the presented stimulus orientation was quantified as the ratio of mean firing rate responses for the presented orientation (X°) to that of the orthogonal to presented stimulus (X + 90°) as described previously. Changes in this measure (and in other visual response properties) were quantified by subtracting CT0 baseline (AM; X°/X + 90°) ratio from CT12 (PM; X°/X + 90°) ratio; this difference was then expressed as a percent change from the baseline value.

### Histology

At the conclusion of each recording, mice were deeply anesthetized with barbiturate injection, and an electrolytic lesion was made at each electrode site (2 mA, 3 s per electrode). Mice were then perfused with formalin and euthanized. Brains were post-fixed, cryosectioned at 50 μm, and stained with DAPI (Fluoromount-G; Southern Biotech) to verify electrode placement in V1.

### Single Unit Identification and Data Analysis

**Figure 1:**
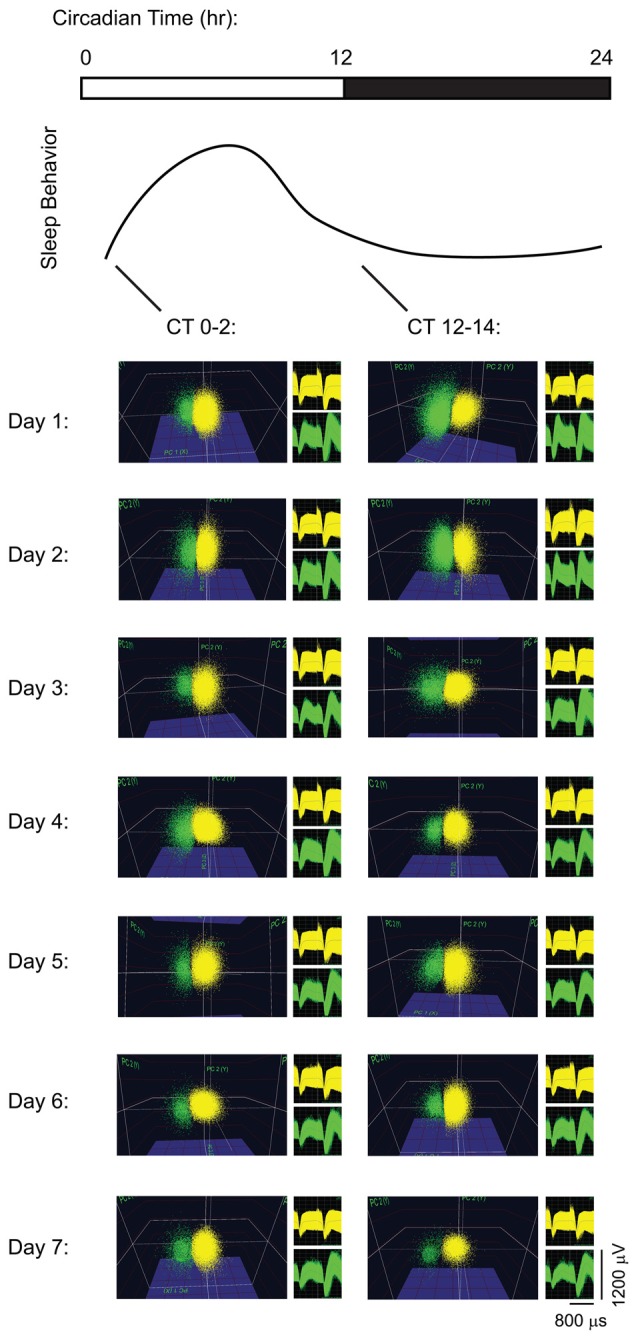
Long-term recordings of primary visual cortex (V1) neurons. Spike data are shown from two representative neurons on a V1 stereotrode across 7 days of continuous recording. For display purposes (i.e., to show stability of spike waveforms over time) neuronal spike data are shown over 2-h intervals of recording time at lights-on (Circadian Time 0 (CT0)–2) and at lights-off (CT12–14) each day, clustered in three-dimensional principal component (PCA) space. Waveforms for the spikes in the two clusters are shown to the right of PCA plots.

Single-neuron data were discriminated offline using standard principle component-based procedures as described previously (Aton et al., 2013, 2014; Ognjanovski et al., 2014, 2017; Durkin and Aton, 2016; Durkin et al., 2017). To ensure stable tracking of single-neuron activity across time, all data analyses were carried out on spike data that was continuously acquired throughout the experiment. Example data are shown for pair of neurons stably recorded on the same V1 stereotrode, from a freely-behaving mouse, in Figure 1. As shown in Figure 1, spikes from individual neurons were discriminated based on consistent spike waveform shape and width, relative spike amplitude on the two stereotrode wires, and relative positioning of waveform clusters in three-dimensional principal component (PCA) space. Single-neuron isolation was verified quantitatively using standard techniques (Hill et al., 2011). Clusters with interspike interval (ISI)-based absolute refractory period violations were eliminated from analysis. Waveform cluster separation (for channels with more than one discriminated single unit) was validated using MANOVA on the first three PCAs (*p* < 0.05 for all sorted clusters), and the Davies-Bouldin (DB) validity index (Sato et al., 2007), as described previously (Durkin et al., 2017; Ognjanovski et al., 2017).

Only neurons that: (1) met the criteria described above and (2) were reliably discriminated and continuously recorded throughout each experiment (i.e., across both 24-h baseline and 12-h experimental condition) were included in firing rate analyses. For analysis of firing rate changes across the population of recorded V1 neurons (e.g., in Figures 3–8), spontaneous and maximal visually-evoked firing rates (calculated as described above) were log_(10)_-transformed. For ANOVA analyses of visual response properties and firing rate changes, all recorded neurons in a given experimental group were grouped into sextiles, based on either their spontaneous firing rate, maximal visually-evoked firing rate, or population coupling strength (see below) at baseline. Sextiling of data allowed statistical comparisons between changes seen in the highest and lowest firing neurons, and direct comparisons of our results with those of other labs (Watson et al., 2016). Changes in firing rate across the day were expressed as a fold change.

**Figure 2:**
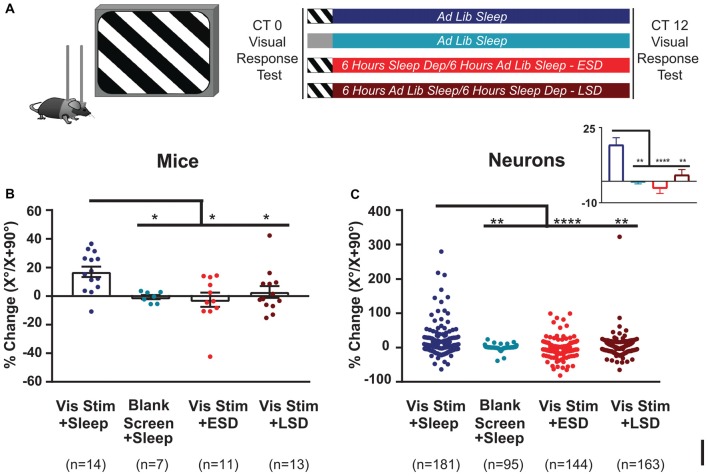
Orientation-selective response potentiation (OSRP) is induced in V1 by visual experience and dependent on subsequent sleep. **(A)** Experimental design. Mice were implanted with stereotrodes to record V1 neurons’ firing across baseline (AM) visual response testing (at lights-on; CT0), 30-min oriented grating stimulus presentation (to induce OSRP) or blank screen presentation, 12 h of subsequent *ad lib* sleep, early sleep deprivation (ESD) or late sleep deprivation (LSD), and a final (PM) visual response test at CT12. Mice were kept in complete darkness (under far-infrared illumination) across CT0–12, to avoid additional visual experience after stimulus presentation. **(B)** OSRP data, showing per-mouse average % changes in neurons’ responses to the presented visual stimulus orientation (X°) vs. the orthogonal orientation (X + 90°); *p* = 0.007, Kruskal-Wallis one-way ANOVA on ranks). Bar graphs show mean ± SEM. Numbers of mice are indicated for each group. **(C)** Neuron by neuron data, as in **(B)** (*p* < 0.0001, Kruskal-Wallis one-way ANOVA on ranks). For all panels, ^*^p < 0.05, ^**^p < 0.01 and ^****^p < 0.0001, Dunn’s *post hoc* test.

**Figure 3:**
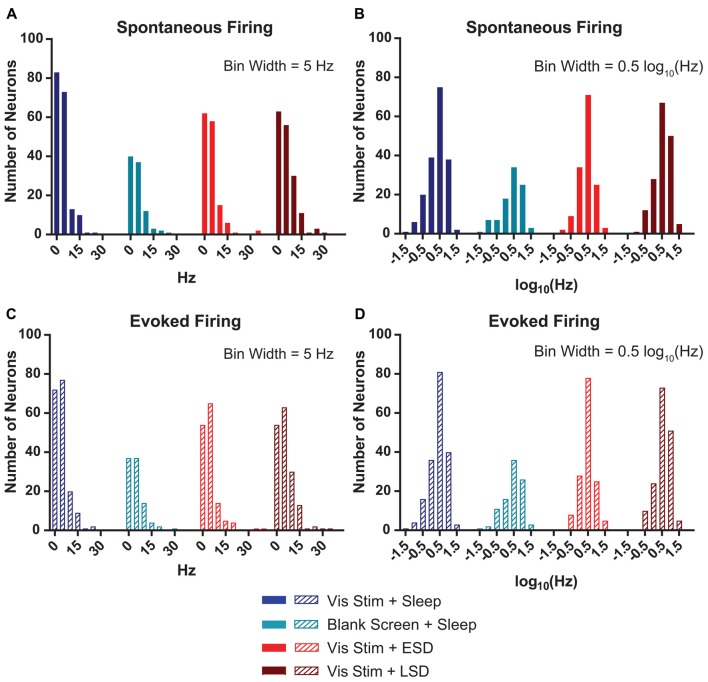
V1 neurons’ spontaneous and evoked firing rates follow a log-normal distribution. **(A)** Distributions of baseline (AM) spontaneous firing rates of the neurons recorded from each of the treatment groups were non-normal (*p* < 0.0001, D’Agostino-Pearson normality test). **(B)** log_(10)_-transformed spontaneous firing rates’ distributions approximated normality (*p* = 0.002, 0.004, 0.15 and 0.02, respectively, for Vis Stim + Sleep, Blank Screen + Sleep, Vis Stim + ESD and Vis Stim + LSD, D’Agostino-Pearson normality test). **(C)** Distributions of baseline (AM) maximal evoked firing rates (i.e., for each neuron’s preferred-orientation stimulus) of the neurons recorded from each of the treatment groups were non-normal (*p* < 0.0001, D’Agostino-Pearson normality test). **(D)** log_(10)_-transformed evoked firing rate data approximated normality (*p* = 0.001, 0.02, 0.55 and 0.05, respectively, for Vis Stim + Sleep, Blank Screen + Sleep, Vis Stim + ESD and Vis Stim + LSD, D’Agostino-Pearson normality test).

**Figure 4:**
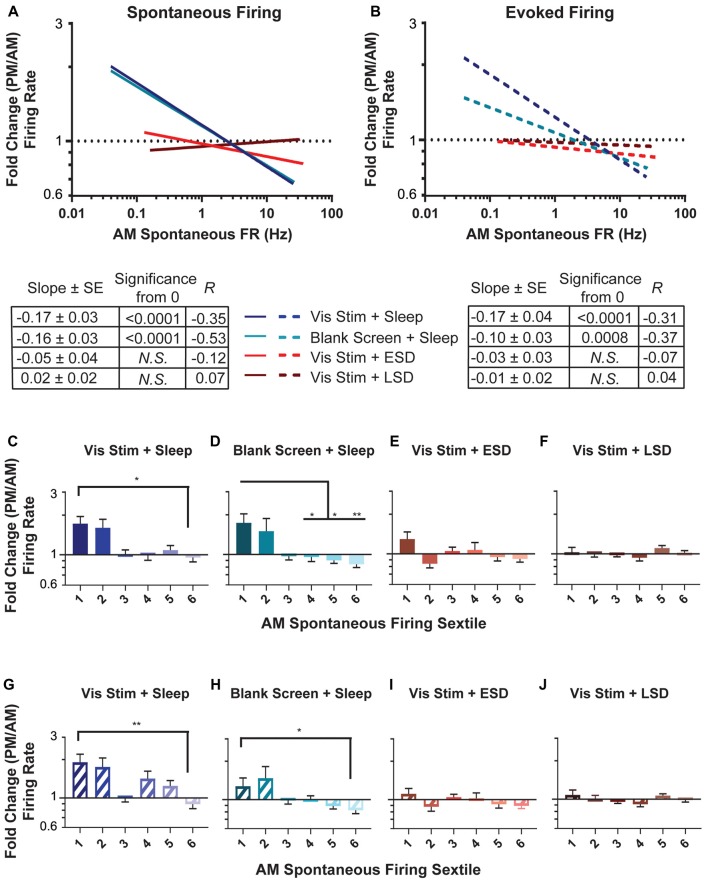
Sleep deprivation impairs neuronal firing rate homeostasis. **(A,B)** Linear fits of data for the change in spontaneous firing rate **(A)** and maximal visually-evoked firing rate (i.e., at each neuron’s preferred stimulus orientation; **(B)** across the day (expressed as a fold change and plotted on a log_(10)_ scale) vs. the AM spontaneous firing rate of the cell (plotted on a log_(10)_ scale). In both groups with *ad lib* sleep, sparsely-firing neurons’ firing rates increased (i.e., showed a fold change >1) while highly active neurons’ firing decreased (i.e., showed a fold change <1). In **(A)** the lines for the visual stimulus and blank screen regressions closely overlap. The table below shows, for each experimental group, the regression slope and SE, Spearman R-value and Bonferroni-corrected *F*-test *p*-value. **(C–F)** Sextiles of the change in spontaneous firing rate, based on AM spontaneous firing rate, which is shown in (**A**; *p* = 0.0015 for panel **(D)** respectively, all others N.S., Kruskal-Wallis one-way ANOVA on ranks). **(G–J)** Sextiles of the change in evoked firing rate, based on AM spontaneous firing rate, which is shown in (**B**; *p* = 0.0356, 0.0087 for panels **(G–H)** respectively, all others N.S., Kruskal-Wallis one-way ANOVA on ranks). Error bars indicate mean ± SEM for log changes in firing rate; ^*^p < 0.05, ^**^p < 0.01, Dunn’s *post hoc* test.

**Figure 5:**
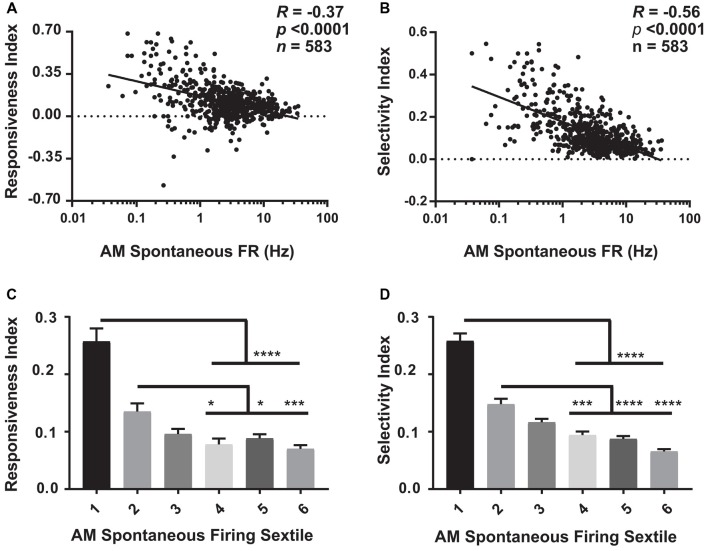
Visual response properties vary across the V1 population as a function of firing rate. **(A)** For baseline (AM) data aggregated across the four experimental groups, there is a significant negative relationship between neurons’ spontaneous firing rate and the responsiveness index (RI; Spearman rank order R- and *p*-values shown). **(B)** A similar negative relationship was seen between AM spontaneous firing rate and neurons’ selectivity index (OSI45; Spearman rank order). **(C,D)** The aggregated data was sextiled based on AM spontaneous firing rate. RI **(C)** and selectivity index (OSI45; **D**) varied significantly across sextiles (*p* < 0.0001, Kruskal-Wallis one-way ANOVA on ranks), with sparsely firing neurons showing higher RI and OSI45 values than faster firing neurons. For panels **(C,D)** ^*^p < 0.05, ^***^p < 0.001, ^****^p < 0.0001, Dunn’s *post hoc* test.

**Figure 6:**
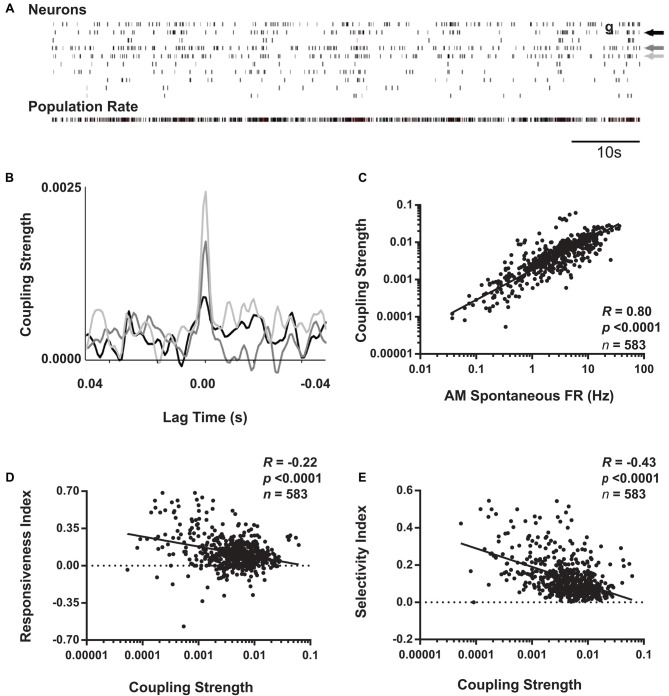
Coupling of V1 neurons’ firing to population activity is negatively correlated with visual responsiveness and orientation selectivity. **(A)** Schematic representation of coupling strength calculation. Across AM visual response testing, spike times for individual neurons (indicated by arrows) were cross-correlated with population activity from all other simultaneously recorded neurons (i.e., with the reference neuron’s activity subtracted from total firing; shown in bottom raster). **(B)** Superimposed cross-correlogram (CCGs) of spiking from the neurons indicated with arrows in the raster plot are shown, following subtraction of the shift-predictor described in “Materials and Methods” section. Coupling strength for each neuron was calculated as the value of the cross-correlation at 0 lag time. **(C)** For baseline (AM) data aggregated from the four experimental groups, coupling strength and spontaneous firing rate show a strong positive correlation. In contrast, at baseline, coupling strength is negatively correlated with both RI **(D)** and OSI45 **(E)**. Spearman rank order R- and *p*-values shown.

**Figure 7:**
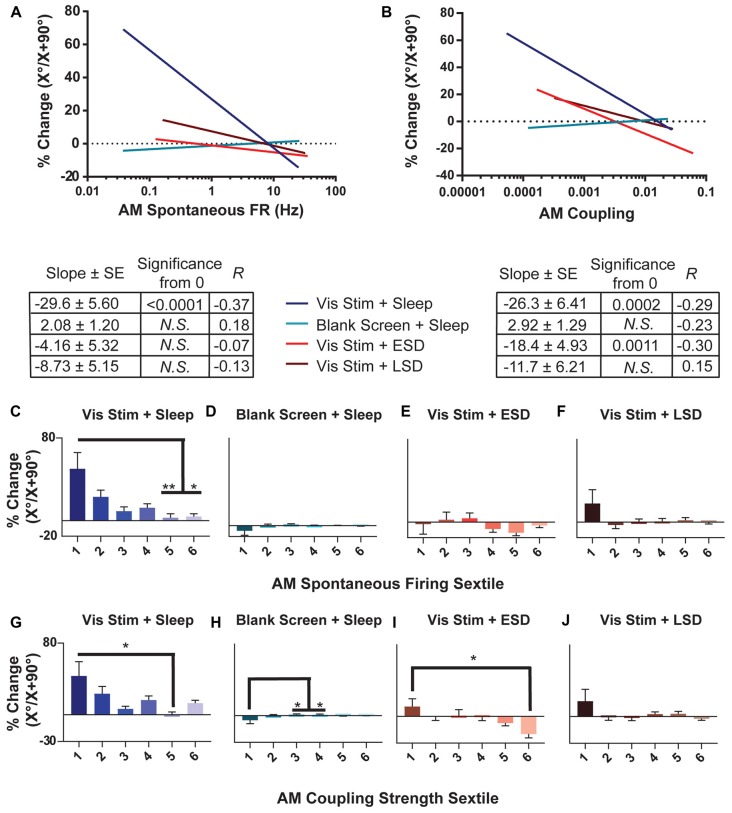
OSRP is greatest in sparsely firing V1 neurons with weak population coupling. **(A,B)** Linear regressions of the relationship between OSRP (expressed as % changes in neurons’ responses to the presented visual stimulus orientation [X°] vs. the orthogonal orientation [X + 90°] across the day, as in Figure 2) and AM spontaneous firing rate. The table below shows, for each experimental group, the regression slope and SE, Spearman R-value, and Bonferroni-corrected *F*-test *p*-value. **(C–F)** Sextiles of the data, based on AM spontaneous firing rate, which is shown in (**A**; *p* = 0.0179 for panel **(C)** all others N.S., Kruskal-Wallis one-way ANOVA on ranks). **(G–J)** Sextiles of the data, based on AM coupling strength, which is shown in (**B**; *p* = 0.0011, 0.0203 for panels **(H–I)** respectively, all others N.S., Kruskal-Wallis one-way ANOVA on ranks). Error bars indicate mean ± SEM for % changes in orientation preference; ^*^p < 0.05, and ^**^p < 0.01, Dunn’s *post hoc* test.

**Figure 8:**
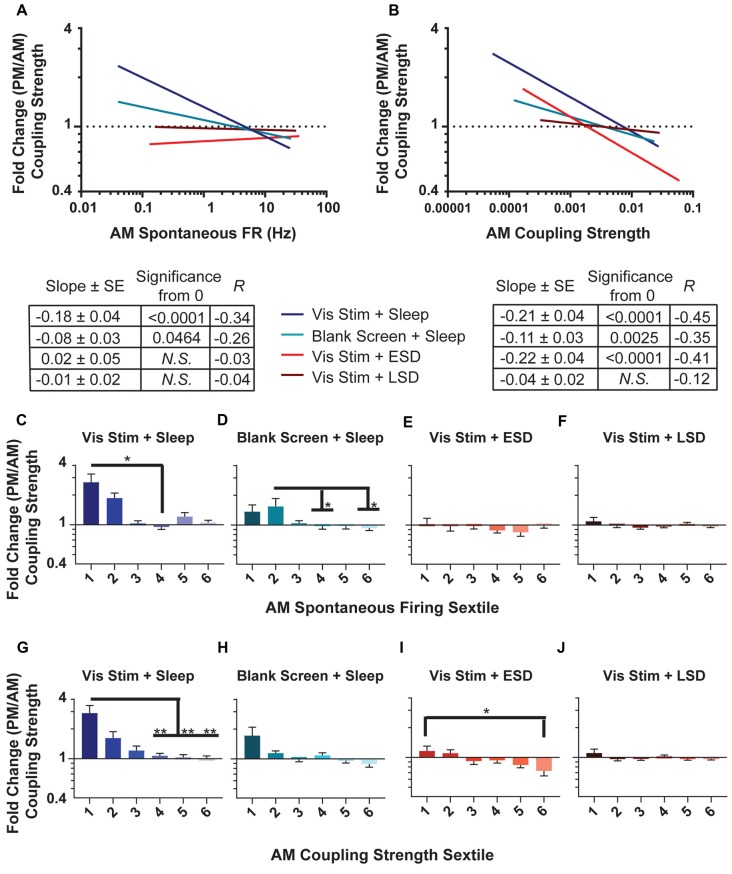
Changes in population coupling strength across the day vary as a function of neurons’ baseline coupling and firing rate, visual experience and sleep. **(A,B)** Linear fits of data for the fold change in coupling strength across the day as a function of AM spontaneous firing rate **(A)** and AM coupling strength **(B)**. The table below shows, for each experimental group, the regression slope and SE, Spearman R-value and Bonferroni-corrected *F*-test *p*-value. **(C–F)** Sextiles of the data, based on AM spontaneous firing rate, which is shown in (**A**; *p* = 0.0043, 0.0391 for panels **(C,D)** respectively, all others N.S., Kruskal-Wallis one-way ANOVA on ranks). **(G–J)** Sextiles of the data, based on AM coupling strength, which is shown in (**B**; *p* = 0.0052, 0.0304 for panels **(G,I)** respectively, all others N.S., Kruskal-Wallis one-way ANOVA on ranks). Error bars indicate mean ± SEM for log changes in firing rate. ^*^p < 0.05, ^**^p < 0.01, Dunn’s *post hoc* test.

Intracortical LFP and nuchal EMG signals were used to categorize recorded data into REM, NREM and wake states over 10-s intervals of recording using custom software. Firing rates were calculated separately within REM, NREM and wake using NeuroExplorer software (Plexon). To assess neuronal firing rate changes across individual bouts of these states, we used a calculation similar to that described previously (Durkin and Aton, 2016). Briefly, firing rates across time were log_(10)_-transformed, and mean firing rates across the first and last 30 s of each state bout were calculated. Changes in firing rate were calculated using custom software, by subtracting the mean firing rate in the first 30 from the mean firing rate in the last 30 s. Bouts with less than 60 s duration, and neurons with a firing rate of 0 Hz in either the first or last 30 s of a particular bout, were excluded from the analysis. Mean rate changes were averaged for all the bouts of a given state (i.e., REM, NREM, or wake) occurring across the entire *ad lib* sleep portion of the experiment for each animal. Thus for Vis Stim + Sleep and Blank Screen + Sleep mice, data were averaged over the CT0 to CT12 *ad lib* sleep recording period; for Vis Stim + ESD mice, data were averaged over the last 6 h of recording; for Vis Stim + LSD mice, data were averaged over the first 6 h of recording.

To assess population coupling, neurons were cross-correlated with the population rate activity during the AM test using a cross-correlogram (CCG) algorithm. Population rate time series were first constructed from the firing of all single units and multi-unit activity across the AM test, with the neuron of interest’s spike times removed; this was done separately for each neuron. Each neuron’s spike train was then cross-correlated with the population rate in 1 ms bins, with counts per bin normalized to the number of reference events as probabilities to account for differences in firing rate. A 95% confidence interval was applied to each CCG. CCGs were corrected using a shift-predictor procedure during the AM and PM tests to correct for coincident firing due to common visually-driven input (similar to methods described in Bachatene et al., 2015) and CCGs were smoothed with a Gaussian filter with a 3 bin width. Peaks in the corrected CCGs were used as measures of population coupling; these peaks were compared between AM and PM tests to assess changes in population coupling across the day.

## Results

### Visual Response Plasticity Among V1 Neurons Relies on Both Visual Experience and Sleep

To characterize effects of visual experience and brain states on visual response properties and firing rates, we first quantified V1 neuronal firing in recordings from C57BL/6J mice. An example showing the stability of our V1 neuronal recordings (from a representative mouse) is shown in Figure 1. Mice underwent continuous recording across a 24-h baseline period (to verify stability of neuronal recordings), a baseline (AM) visual response assessment (at lights-on; CT0), a 30-min presentation of a single oriented flickering grating (or as a negative control, a blank screen), and a 12-h post-stimulus period in complete darkness (to prevent additional visual experience). During this post-stimulus period, mice were either allowed *ad lib* sleep, or were sleep deprived by gentle handling over the first or last 6 h (ESD or LSD). At CT12, response properties were reassessed to quantify OSRP and other changes in visual responses (Figure 2A). As we have shown previously (Aton et al., 2014; Durkin and Aton, 2016; Durkin et al., 2017), oriented grating presentation resulted in an increase among V1 neurons’ firing rate responses to the presented stimulus orientation. Consistent with our prior findings, both ESD and LSD disrupted OSRP. This was true for both the average OSRP of each mouse (i.e., measured across all neurons recorded from each mouse; Figure 2B) and for all neurons recorded in a given condition (Figure 2C). As we have shown previously, there were no significant differences between OSRP measurements for male vs. female mice(Durkin et al., 2017), different neuronal subclasses (i.e., principal neurons vs. fast-spiking interneurons; Aton et al., 2014), or differential timing of sleep deprivation (Aton et al., 2014).

### Spontaneous and Visually-Evoked Firing Among V1 Neurons Approximates a Lognormal Distribution

Watson et al. (2016) recently demonstrated that mean firing rates of frontal cortical neurons show a roughly lognormal distribution. For our V1 recordings, we calculated the baseline (AM) spontaneous firing rate during blank screen presentation. We found that, as is true in frontal cortex, the distribution of spontaneous firing rates shows a clearly non-normal distribution (*p* < 0.0001 for all experimental groups, D’Agostino-Pearson normality test—Figure 3A). As shown in Figure 3B, when neuronal firing rates are log_(10)_-transformed, although most groups’ distributions remain statistically non-normal, each is a closer approximation of normality (Vis Stim + Sleep: *p* = 0.002, Blank Screen + Sleep: *p* = 0.004, Vis Stim + ESD: *p* = 0.15, Vis Stim + LSD: *p* = 0.017, D’Agostino-Pearson normality test). A similar pattern was seen for distributions of maximal visually-evoked firing rates (i.e., for responses to each neuron’s preferred stimulus orientation; Figures 3C,D). Thus spontaneous and visually-evoked firing rate data were log_(10)_-transformed for all subsequent analyses.

### Sleep Promotes, and Sleep Deprivation Impairs, Re-distributions of Firing Rates Among V1 Neurons

We next assessed how sleep changes firing rates across the V1 neuronal population. As shown in Figure 4, neurons recorded in both *ad lib* sleep conditions (following either visual stimulus or blank screen presentation) showed a re-distribution of both spontaneous (Figure 4A) and maximal visually-evoked (Figure 4B) firing rates across the day. This re-distribution was systematic, in that (as is true for firing changes across sleep in frontal cortex; Watson et al., 2016), the lowest firing neurons showed increases in firing rate, and the highest firing neurons showed decreases in firing rate. This is illustrated by taking the regression of (log_(10)_-transformed) baseline (AM) spontaneous firing compared with the fold change in firing across the day. In the absence of systematic firing changes across the baseline V1 firing rate distribution, one would expect the slope of this regression to be zero. We find that firing rate changes among neurons in both *ad lib* sleep conditions (i.e., regardless of the type of visual experience) show negative relationships to baseline spontaneous firing, which are significantly different from zero (Vis Stim + Sleep: *p* = 0.003, Blank Screen + Sleep: *p* < 0.001 Spearman rank order, *F*-test *p* < 0.001). In contrast to what is seen in V1 of non-sleep deprived mice, V1 neurons recorded across both early and late sleep deprivation (ESD and LSD) conditions showed no systematic firing rate changes (for either spontaneous or visually-evoked firing rates). This is shown in Figures 4A,B, where for ESD and LSD, the regressions of neurons’ firing rate changes vs. their baseline firing rates do not differ from zero (*N.S*., *F-test*).

Recent work (Watson et al., 2016) assessed sleep-dependent firing rate changes among neurons that had been grouped into sextiles based on their mean firing rates. We carried out a similar analysis on V1 neurons’ firing rate changes. As shown in Figures 4C–J, in mice from both sleeping conditions, across-the-day firing rate changes varied in V1 depending on baseline firing rate sextile. For both spontaneous (Figures 4C,D) and maximal visually-evoked (Figures 4G,H) firing, the lowest-firing sextile of V1 neurons from the two sleeping conditions (regardless of visual experience) showed firing rate increases relative to the highest-firing sextile, where neurons tended to have firing rate reductions across the day. This effect was not present in either of the two sleep deprived groups (ESD and LSD), where both spontaneous (Figures 4E,F) and visually-evoked (Figures 4I,J) firing rate changes across the day did not vary as a function of baseline firing rate. Together, these analyses suggest that firing rates in V1 neurons are altered across a day of *ad lib* sleep, as a function of their baseline firing rate, and that sleep deprivation (at any time of day) disrupts this process.

### V1 Neurons’ Visual Response Properties Vary as a Function of Baseline Firing Rate

Our analyses of firing rates suggest that specific subpopulations of V1 neurons (those with the lowest and highest baseline firing rates) undergo the largest sleep-dependent alterations in firing (increases and decreases in firing rate respectively). One question, in light of the well-described effects of sleep on visual response plasticity (Frank et al., 2001; Aton et al., 2009a, 2013, 2014; Durkin and Aton, 2016; Durkin et al., 2017), is how visual response properties vary between sparsely firing and higher firing neurons. We assessed how visual responses varied at baseline (i.e., during the AM visual response test at CT0) as a function of firing rate. As shown in Figure 5 (where baseline [AM] data from the four experimental groups are aggregated), we found that both visual responsiveness (Figure 5A) and orientation selectivity (Figure 5B) are highest for sparsely firing neurons, and show a significant negative relationship with spontaneous firing rate. This relationship was statistically significant (*p* < 0.0001, Spearman rank order) and regressions were significantly different from 0 (*p* < 0.0001, *F*-test) across all four experimental groups (when examined separately). A similar relationship between spontaneous firing rate and visual response properties was seen during the CT12 (PM) test (*p* < 0.0001, Spearman rank order; *p* < 0.0001 for slope significance from 0, *F*-Test). Figures 5C,D show that these properties vary significantly by firing rate sextile. Together, this suggests that those V1 neurons that show sleep-associated increases in firing (i.e., the lowest-firing neurons) are highly visually responsive and sharply orientation-tuned, and thus encode highly specific visual information. Conversely, V1 neurons that show sleep-associated firing decreases (i.e., the highest-firing neurons) are less visually responsive and less orientation selective.

### V1 Neurons’ Visual Response Properties Vary With Population-Coupling Strength

Recent studies have categorized populations of neurons in sensory cortex, not based on firing rate, but rather on how strongly coupled their firing is to population activity (Bachatene et al., 2015; Okun et al., 2015). Okun et al. (2015) and Bachatene et al. (2015) classified cortical neurons into “choristers” (i.e., strongly coupled) and “soloists” (i.e., weakly coupled) based on how correlated their firing was with population activity during both visual stimulation and spontaneous activity. We similarly calculated coupling values for each neuron as the peak of the CCG between each spike train and the population rate summed from all other neurons recorded simultaneously (Figures 6A,B). Similar to results seen by Bachatene et al. (2015) there was a significant relationship between spontaneous firing rate and population coupling (Figure 6C), where highly-coupled neurons (“choristers”) exhibited higher spontaneous firing rates (*p* < 0.0001, Spearman rank order).

We next examined how baseline visual response properties varied as a function of how strongly coupled neuronal firing is to V1 population activity. We found that across all groups, coupling strength showed a significant negative relationship to both visual responsiveness and orientation selectivity at baseline (Figures 6D,E). These findings are consistent with previous literature demonstrating that weakly coupled neurons tend to encode more specific visual information, while strongly coupled neurons do not (Bachatene et al., 2015; Okun et al., 2015). However, we also found that this relationship was likely mediated by differences in baseline spontaneous firing rates among neurons (*p* = 1e-8 and *p* = 3e-5, respectively, Sobel tests for mediation of the relationships between population coupling and visual responsiveness and between population coupling and orientation selectivity).

### OSRP Varies Across the V1 Population, as a Function of Both Baseline Firing Rate and Population Coupling

To characterize how experience- and sleep-dependent plasticity varies across the population of V1 neurons, we next characterized changes in orientation preference across the day based on neurons’ initial firing rate and population coupling. As shown in Figure 7, we found that among neurons recorded from non-sleep deprived mice, OSRP was greatest among neurons with the lowest baseline firing rates. The magnitude of OSRP was negatively correlated with baseline firing rate in mice allowed *ad lib* post-stimulus sleep (Vis Stim + Sleep; *p* = 0.0375, Spearman rank order), but critically, showed no relationship to baseline firing rate in Blank Screen + Sleep, Vis Stim + ESD, or Vis Stim + LSD mice (*N.S*., Spearman rank order; Figure 7A). When neurons’ spontaneous AM firing rates were grouped into sextiles (Figures 7C–F), the lowest-firing sextile showed significantly greater OSRP than neurons in the highest-firing sextiles for mice allowed *ad lib* sleep. However, OSRP was similar in magnitude across baseline firing sextiles in both sleep deprived groups. Similarly, the baseline coupling of firing to population activity tended to be a good predictor of the magnitude of OSRP across the day in neurons recorded from Vis Stim + Sleep mice and Vis Stim + ESD mice (where weakly-coupled neurons showed significantly greater OSRP than strongly-coupled neurons), but not from Blank Screen + Sleep and Vis Stim + LSD mice (Figures 7B,G–J). The relationship between population coupling and OSRP appeared to be mediated in part by baseline firing rate among neurons recorded from the Vis Stim + Sleep group (*p* = 1e-6, Sobel test). However, the same was not true for neurons recorded from Vis Stim + ESD mice, where firing rates did not predict OSRP. These data show that experience-dependent plasticity is not expressed uniformly across the V1 population, but is greatest among sparsely firing and weakly population-coupled neurons after a period of subsequent sleep.

**Figure 9:**
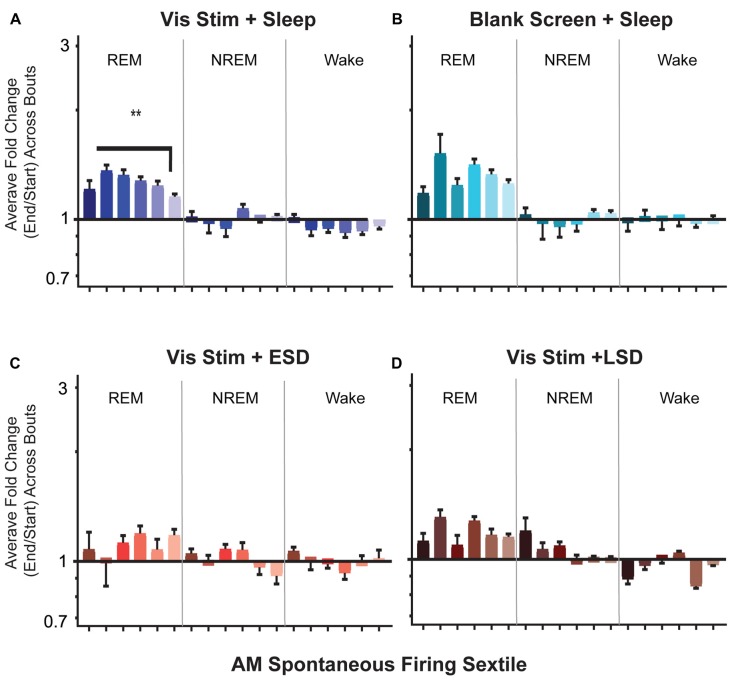
Firing rates of V1 neurons increase across bouts of rapid eye movement (REM) sleep. **(A–D)** Neuronal firing rates were averaged over the first and last 30 s of each REM sleep, non-REM (NREM) sleep, or wake bout, and average firing rate changes across the portion of the day corresponding to *ad lib* sleep were calculated for each neuron (see “Materials and Methods” section). Values indicate mean ± SEM for state specific changes in firing for each sextile of baseline (AM) spontaneous firing rate (sextile colors as in Figures 4, 7). While firing rates were minimally affected across periods of NREM and wake, in the Vis Stim + Sleep group **(A)**, increases in firing across post-stimulus REM bouts varied as a function of baseline (AM) spontaneous firing rate (*p* = 0.0069, Kruskal-Wallis one-way ANOVA on ranks). ^**^p < 0.01, Dunn’s *post hoc* test. While a similar trend was seen for Blank Screen + Sleep **(B)**, there was no statistically significant effect of baseline firing rate. Changes in firing across REM were not statistically significant during the 6 h of recovery sleep in Vis Stim + ESD mice **(C)**, or over the first 6 h of *ad lib* sleep in Vis Stim + LSD mice **(D)**.

### V1 Neurons’ Population-Coupling Strength Is Altered by Visual Experience and Sleep

Because population coupling could itself be altered as a function of circuit plasticity, we next assessed how the strength of population coupling changes across the day for different neuronal populations. AM and PM population coupling were highly correlated across all groups (*R* = 0.82, 0.94, 0.85, 0.64 for Vis Stim + Sleep, Blank Screen + Sleep, Vis Stim + ESD and Vis Stim + LSD, respectively; all *p* < 0.000001, Spearman rank order). However, there was a significant increase in coupling from AM to PM time points in the Vis Stim + Sleep condition (*p* = 0.014; Wilcoxon signed rank test) and significant decrease in coupling from AM to PM in the Vis Stim + ESD condition (*p* = 0.001; Wilcoxon signed rank test). These changes were not uniform, but instead varied across the distribution of both V1 neurons’ baseline (AM) spontaneous firing rates (Figure 8A) and their baseline (AM) population coupling strength (Figure 8B). Spontaneous firing rates were predictive of across-the-day coupling strength changes for neurons recorded from both sleeping groups (*p* < 0.003 and *p* < 0.005 for Vis Stim + Sleep and Blank Screen + Sleep respectively, Spearman rank order, Figure 8A), with lower-firing neurons showing the greatest increase in coupling strength across the day (Figures 8C,D). There was no relationship between baseline firing rate and coupling strength changes for neurons recorded from Vis Stim + ESD and Vis Stim + LSD mice (Figures 8A,E,F). Baseline population-coupling strength was predictive of coupling strength changes in three of the four experimental groups following visual stimulus presentation (Vis Stim + Sleep, Blank Screen + Sleep and Vis Stim + ESD; all *p* < 0.005; Vis Stim + LSD *N.S*., Spearman rank order, *F*-test), with neurons with the lowest coupling strength at baseline showing the largest increases in coupling strength across the day (Figure 8B). In spite of the maintained correlation in the Vis Stim + ESD group, the net change in coupling is negative, in contrast to the range of changes in the sleep conditions (Figures 8G–J).

### Firing Rates of V1 Neurons Are Differentially Altered Across Bouts of NREM, REM and Wake

We next examined how firing rates among V1 neurons are altered across individual bouts of NREM, REM and wake. Across groups, we found V1 firing changed little across NREM or wake bouts. In contrast, in both Vis Stim + Sleep and Blank Screen + Sleep mice, neurons showed an apparent increase in firing across bouts of REM (Figure 9). In Vis Stim + Sleep mice, as was true for firing increases across the day in these mice, this effect of REM was not uniform, but preferentially affected neurons with lower baseline firing rates (Figure 9A). There was a similar trend across REM for neurons in Blank Screen + Sleep mice, although this did not reach statistical significance (Figure 9B). There were no significant differences between sextiles in either sleep deprivation condition (Figures 9C,D). When overall changes in firing rates across REM sleep were compared between groups, Vis Stim + Sleep and Blank Screen + Sleep showed larger total changes in firing rates than either sleep deprivation group (*p* < 0.0001, Kruskal-Wallis one-way ANOVA on ranks; Vis Stim + Sleep or Blank Screen + Sleep vs. Vis Stim + ESD, *p* ≤ 0.001; Vis Stim + Sleep or Blank Screen + Sleep vs. Vis Stim + LSD, *p* < 0.0001, Dunn’s *post hoc* test). A regression of sextile averages across 2 h time blocks between CT0 and CT12 showed no significant modulation of firing changes during REM bouts by time of day in the Vis Stim + Sleep group. This suggests that REM bout-associated firing increases may be similar in magnitude across the entire rest phase following visual experience.

## Discussion

We have previously shown that, following a period of patterned visual experience, sleep facilitates visual response changes (OSRP) among mouse V1 neurons (Aton et al., 2014; Durkin and Aton, 2016; Durkin et al., 2017). While visual responses are not altered across waking exposure to an oriented grating, after a 12-h period of subsequent sleep, firing rate responses to gratings of the same orientation are selectively enhanced (Durkin and Aton, 2016). This selective enhancement of firing rate responses is disrupted by post-stimulus sleep deprivation (Aton et al., 2014; Durkin and Aton, 2016; Durkin et al., 2017). The underlying mechanisms for OSRP expression appear to involve thalamocortical long-term potentiation (LTP), as OSRP and LTP are mutually occluding *in vivo* (Cooke and Bear, 2010) and rely on similar intracellular signaling pathways (Frenkel et al., 2006). We have also recently shown that across visual experience (while V1 responses are unaffected), visual responses to the presented stimulus orientation are selectively enhanced in the LGN (Durkin et al., 2017). This suggests that information content regarding prior visual experience (i.e., orientation-specific information) is relayed from thalamus to cortex during post-stimulus sleep. Here, we aimed to clarify how this information is distributed among neurons in the sensory cortex, and how this relates to what is known about the heterogeneity of neuronal firing rates, population coupling and sleep-dependent changes in firing (Bachatene et al., 2015; Okun et al., 2015; Watson et al., 2016). We find that sleep-dependent OSRP is not uniform across the population of V1 neurons. Rather, it is expressed preferentially among sparsely firing V1 neurons. These neurons are weakly coupled to V1 population activity (i.e., they are “soloists” rather than “choristers”) are more visually responsive than other V1 neurons, and have greater orientation selectivity than neighboring neurons. These neurons also selectively show firing increases across sleep—a process that (like OSRP itself; Aton et al., 2014) is disrupted by partial sleep deprivation. Intriguingly, this same population of neurons also becomes more strongly coupled to population activity across a period of sleep.

Our present data suggest that for sensory cortical areas, the heterogeneous firing rate changes previously reported in frontal cortex across sleep (i.e., increases in firing among sparsely firing neurons, and simultaneous reductions in firing among high firing neurons; Watson et al., 2016) may have special functional significance. By preferentially augmenting firing in neurons that show the highest responsiveness and selectivity, sleep may function generally to increase the signal-to-noise ratio of sensory responses. This is particularly relevant after an experience that induces response plasticity in the cortex, such as after visual experience that induces OSRP in V1. This idea is reminiscent of predictions of the “synaptic homeostasis hypothesis (SHY),” which proposes that sleep may improve signal-to-noise ratios in the spiking of neural circuits through firing reductions caused by general synaptic downscaling (Tononi and Cirelli, 2003; Hill and Tononi, 2005; Cirelli and Tononi, 2014). While our present findings do not address the synaptic basis of these changes, we find that improvements in sensory signal-to-noise ratios may be caused by simultaneous increases and decreases in the firing of distinct neuronal populations during sleep.

The fact that these firing rate changes are disrupted by sleep deprivation (either ESD or LSD) suggest that the mechanism underlying these heterogeneous changes in neuronal firing rate is distinctly sleep-dependent. This is supported by our analysis of firing rate changes across bouts of REM, NREM and wake, where we find increases in firing rates, which are greatest in more sparsely firing neurons, occurring preferentially across periods of REM. This is in line with our prior work, showing firing rate increases in V1 neurons across REM bouts in the hours after visual stimulus presentation, but not after presentation of a blank screen (Durkin and Aton, 2016). The fact that we also see increases across REM bouts in our blank screen condition in this study is likely due to the fact that we are assessing firing rate changes across the entire day (not over the first few hours following visual experience, as in the prior study). Because REM preferentially affects the activity of sparsely firing V1 neurons, this brain state may account for the firing increase seen across the day in this population. Intriguingly, this phenomenon seems to be exactly the opposite of changes in firing seen across REM in the hippocampus (Grosmark et al., 2012; Miyawaki and Diba, 2016), and frontal cortex (Watson et al., 2016), where neuronal firing decreases across the population.

An unanswered question is what mechanism could mediate differential changes in the firing rates of sparsely firing and high firing neuronal populations across a period of sleep. A number of potential physiological mechanisms, regulated by activity patterns present in thalamocortical circuits during sleep, may explain these apparent simultaneous reductions and enhancements of firing in different neuronal populations (Puentes-Mestril and Aton, 2017; Roach et al., 2018). One prominent hypothesis proposes that neurons activated by waking experience are preferentially re-activated during subsequent sleep, in the context of sleep-associated oscillations (Huber et al., 2004; Aton, 2013; Batterink et al., 2016; Antony et al., 2018). Thus it is possible that lower-firing neurons are preferentially re-activated during sleep, while higher-firing neurons are not. This could lead to differential activity-dependent plasticity (and thus firing changes) in sparsely firing and higher firing populations across a period of sleep. While our present analyses do not specifically test this mechanism, our previous studies of OSRP have shown that V1 and LGN neurons that exhibit the most coherent firing during NREM oscillations show the most dramatic changes in responsiveness to the presented stimulus orientation (Aton et al., 2014; Durkin et al., 2017). Another possibility is that, because high-firing neurons neurons in this study likely include fast-spiking interneurons, the firing decreases seen after sleep among higher-firing neurons are due to differential effects of sleep on excitatory and inhibitory neuronal populations. This would be not be an unprecedented finding—in previous recordings of rat cortical neurons, Vyazovskiy et al. (2009) reported significant firing rate decreases across sleep only in physiologically-defined fast spiking interneurons. We and others have speculated previously that suppression of activity in the fast-spiking interneuron population may serve as a critical mechanism for some forms of sleep-dependent plasticity (Aton et al., 2013; Puentes-Mestril and Aton, 2017). One intriguing possibility, worthy of future study, is that firing rate increases seen across a period of sleep in sparsely-firing neurons are the direct result of disinhibition.

Regardless of the mechanisms underlying the heterogeneous changes we observe in V1 neurons’ firing rate and population coupling, the nature of these changes is likely to be highly informative for promoting visual response plasticity. We find that after a period of uninterrupted sleep, the most highly visually-responsive and orientation-selective neurons show increased firing, while less responsive and more poorly-tuned neurons show decreased firing. Moreover, we find that following patterned visual experience (which induces response plasticity), these highly responsive and selective neurons preferentially increase the coupling of their firing to population activity. Together, these data suggest that in the context of sleep-dependent sensory plasticity, neurons which carry highly specific visual information have an increased capacity to influence population activity in V1.

## Author Contributions

BC, JD and SA designed research and wrote the article. AS and SA performed research. CB contributed new analytic tools. BC, JD, EP and SA analyzed data.

## Conflict of Interest Statement

The authors declare that the research was conducted in the absence of any commercial or financial relationships that could be construed as a potential conflict of interest.
